# Tristatic observation of polar mesosphere winter echoes with the EISCAT VHF radar on 8 January 2014: a case study

**DOI:** 10.1186/s40623-018-0878-5

**Published:** 2018-07-03

**Authors:** Evgenia Belova, Maria Kawnine, Ingemar Häggström, Tima Sergienko, Sheila Kirkwood, Anders Tjulin

**Affiliations:** 1 Swedish Institute of Space Physics, Kiruna, Sweden; 2 Motionlogic GmbH, Berlin, Germany; 3 EISCAT Scientific Association, Kiruna, Sweden

**Keywords:** PMWE, EISCAT, Tristatic radar measurements, Mesospheric winds and wind shear, Ducted gravity waves

## Abstract

Polar mesosphere winter echoes (PMWE) were observed at 70 km over Tromsø, Norway, on 8 January 2014 using the tristatic configuration of the European incoherent scatter VHF radar. For the interval 11:00–13:00 UT where the strongest patch of PMWE of about 6-min duration was detected, the spectra of the received signal were analysed for the Tromsø site and altitude profiles of spectral parameters were derived. For the remote sites Kiruna and Sodankylä, the Doppler velocities and their vertical shear were determined by using the measured autocorrelation functions. Ducted gravity waves with periods of 5–10 min were found in the vertical wind velocity between 66 and 81 km altitudes. The duct might be formed around 70 and 77 km altitude where horizontal wind maxima were observed with the Kiruna receiver. However, we did not find any close relation between wind shear at 70 km altitude and PMWE at the same height: the wind shear was present for 2 h, but PMWE for only 6 min. Enhanced spectral width in the vertical Tromsø beam was observed for the PMWE patch. We discussed these experimental findings in relation to the winter echo generation mechanism. Our conclusion is that the presence of patchy negatively charged small-sized dust might explain the observations although a gravity wave breaking mechanism cannot be completely rejected.
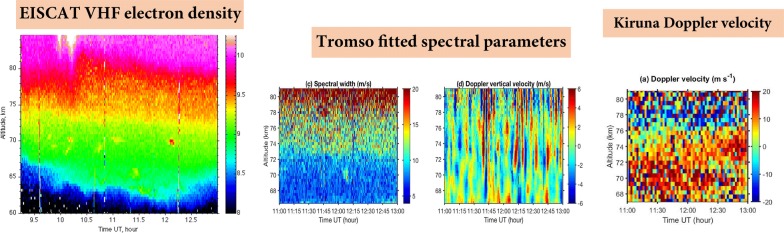

## Introduction

Polar mesosphere winter echoes (PMWE) are enhanced backscatter from 50 to 80 km altitudes observed by VHF radars in the winter and equinox seasons in polar regions. They, on average, are an order of magnitude weaker and occur at lower altitudes than polar mesospheric echoes in summer (PMSE), although in some cases PMWE can be as strong as weaker PMSE (Kirkwood et al. 2013, 2015). Powerful radars, e.g. the MAARSY radar in Andenes, Norway, can regularly observe even weak winter echoes, and a PMWE mean occurrence rate of 16% for 2011–2013 has been reported by Latteck and Strelnikova (2015). In order to see PMWE with other radars, such as the ESRAD mesosphere-stratosphere-troposphere radar at Esrange, Sweden, additional ionisation of the lower ionosphere is needed. Elevated electron density during solar proton events provides good conditions to observe winter echoes (e.g. Kirkwood et al. 2002; Belova et al. 2013). Enhanced electron precipitation from the magnetosphere during high-speed solar wind streams has also been found to give favourable conditions for PMWE occurrence (Kirkwood et al. 2015).

PMWE have been observed with atmospheric radars in the Arctic at about 50 MHz as well as with the European incoherent scatter (EISCAT) VHF radar at 224 MHz (e.g. Czechowsky et al. 1979; Ecklund and Balsley 1981; Belova et al. 2005; Kirkwood et al. 2006a; Zeller et al. 2006; Strelnikova and Rapp 2013). They have also been observed over Antarctica (Morris et al. 2011; Kirkwood et al. 2015; Nishiyama et al. 2015).

The mechanism of PMWE generation is still unknown; however, a few hypotheses have been proposed. Lübken et al. (2006) suggested that at least some PMWE can be explained by layered neutral turbulence excited by wind shear. Rapp et al. (2011) using the MAARSY radar in multi-beam experiment showed a relation of PMWE with gravity wave breaking that generates turbulence. In contrast, Stebel et al. (2004) and Belova et al. (2005) claimed that turbulence alone cannot account for the observed echo strength. It was suggested that small charged dust particles are involved (Kirkwood et al. 2002, 2006a, 2015). The presence of such dust, probably meteoric smoke particles, during PMWE has been confirmed in so-called heating—overshoot experiments, where the ionosphere is modified by a powerful RF-wave from the ground (Belova et al. 2008; La Hoz and Havnes 2008). High-speed horizontal movement of the scattering location reported by Kirkwood et al. (2006b) in relation with strong PMWE have evoked a hypothesis of their non-turbulent origin involving infrasound waves coming from below and probably originated from ocean swell (Le Pichon et al. 2009).

Because one of the key parameters for understanding PMWE generation mechanism is horizontal velocity of scatterers, it is important to measure it and estimate wind shear. For the ESRAD radar, we can derive horizontal velocities for very strong PMWE only using full correlation analysis. Another possibility is to use the EISCAT VHF radar in a tristatic experiment when the radar wave is transmitted from one site near Tromsø, Norway and received at three sites Tromsø, Kiruna (Sweden) and Sodankylä (Finland). Such an experiment was recently used for a PMSE study, where wind shear was observed in the layer of enhanced radar echo (Mann et al. 2016).

There are few tristatic EISCAT radar observations of PMWE so far because strong, long-lasting PMWE are quite rare phenomena and the EISCAT radars do not operate continuously (with a few exceptions) but on a campaign/experiment basis, i.e. according to a predefined schedule. Thus, even when PMWE are present (as one can judge using 50 MHz atmospheric radars operating continuously), it is not always possible to start the EISCAT experiment at short notice.

In this paper we will analyse PMWE measurements made on 8 January 2014 when the tristatic EISCAT VHF radar was operated during about 4 h. We aim to derive horizontal and vertical winds and analyse whether the winds and other background atmospheric conditions are related to the PMWE occurrence. We also compare spectral characteristics of PMWE with those of normal incoherent scatter (IS). The paper is organised as follows. Firstly, we describe the experiment setup and data analysis. Then the experimental results will be presented followed by discussion. Conclusions including suggestions of the PMWE generation mechanism for the event under consideration will finalise the paper.

## Experiment description

Since 2012 the EISCAT VHF radar can be used in a tristatic configuration. It transmits/receives at the EISCAT site near Tromsø and receives at two sites: one near Kiruna and other one near Sodankylä. The distances between Tromsø (TRO) and the remote sites are 199 and 391 km for Kiruna (KIR) and Sodankylä (SOD), respectively.

**Fig. 1:**
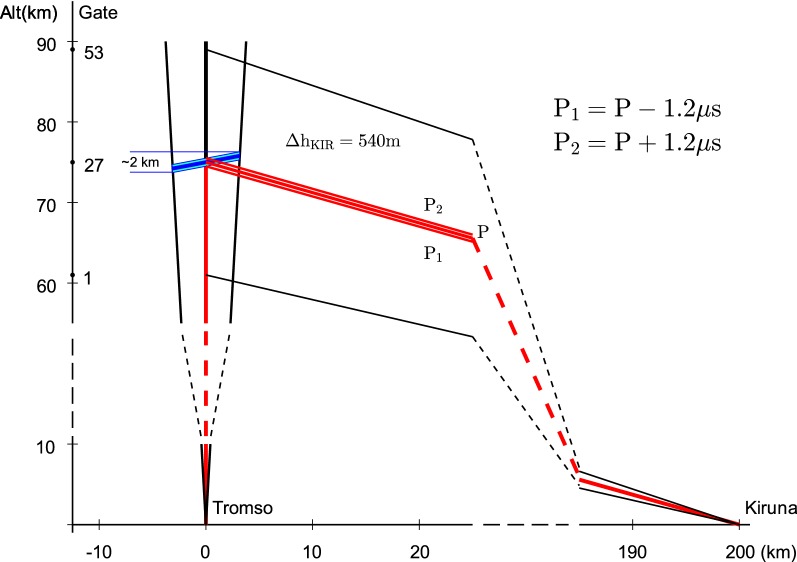
Sketch of the experiment geometry in the vertical plane between Tromsø-Kiruna (not to scale). Transmitting vertically at TRO and receiving at KIR site. KIR receiver is pointed to 75 km over TRO and samples scattered signal in 53 range gates (travel times) with 2.4 μs separation. The travel paths (times) *P*_1_, *P*_2_ and *P* indicating the lower and upper boundaries and the middle, respectively, for the central gate 27 are marked in red. The positions of the scatterers, which contribute to the signal at gate 27, are marked in blue. See the text for more information

On 8 January 2014 from 9:15 to 13:00 UT the EISCAT VHF radar ran the “manda” experiment (experiment and radar parameters are shown in Table 1). The TRO beam was pointed vertically; KIR and SOD receivers were elevated by 19.6° and 9°, respectively, pointing at 75 km altitude above Tromsø. At the remote sites, the scattered signal was sampled in 53 range gates (travel times) centred at the pointing altitude and separated by 2.4 μs (i.e. 26 range gates with travel time shorter than for signal scattered from 75 km altitude and 26 range gates with travel time longer than that). Geometrical calculations give the maximum and minimum heights (*H*_max_ and *H*_min_) seen by KIR and SOD receivers, as well as the altitude resolution (see Table 1). In reality, one should take into account transmit beam width which makes the situation more complex. A sketch of the geometry of the experiment is shown in Fig. 1. At each sampling time, the remote site (e.g. KIR) receives signals from all paths which have the same length: TRO-scattering volume-KIR. Such points lie on the surface of a prolate spheroid with the foci at TRO and KIR, the parameters of which can be calculated. In the figure, the path P for the range gate centred at 75 km altitude as well as paths P_1_ and P_2_ for the lower and upper border of the gate are shown. The illuminated volume is approximated by a cylinder with diameter of 3.1 km corresponding to half-power beam width of 2.4°. We estimated that at this range gate the signal comes from altitudes 74–76 km with major contribution from 75 km ± 0.27 km. This issue of intersecting beams will be a subject of future detailed investigation elsewhere.

**Table 1:** Experiment parameters (only used in the calculations) and radar parameters

	Tromsø	Kiruna	Sodankylä
Geographic coordinates	69°35′N 19°14′E	67°52′N 20°26′E	67°22′N 26°38′E
Frequency	224.4 MHz		
Peak power	1.5 MW		
Half-power beam width	1 × 2.4° × 1.7° T_*x*_ 2 × 2.4° × 1.7° R_*x*_	1.2°	1.2°
Azimuth		346.3°	312.7°
Elevation	90°	19.6°	9°
Bragg scale	0.67 m	0.82 m	0.88 m
Pointing height		75 km	75 km
Number of range gates	250 (for D region)	53	53
Altitude resolution Δ*h*	360 m	540 m	590 m
Minimum height (*H*_min_)	19 km	61 km	59.4 km
Maximum height (*H*_max_)	109 km	89 km	91 km

For the D region, we used pulse-to-pulse correlation to build an autocorrelation function (ACF) of 127 lags with increment of 1.5 ms. The pulse baud length is 2.4 μs resulting in 360 m range resolution for TRO.

## Data analysis

**Fig. 2:**
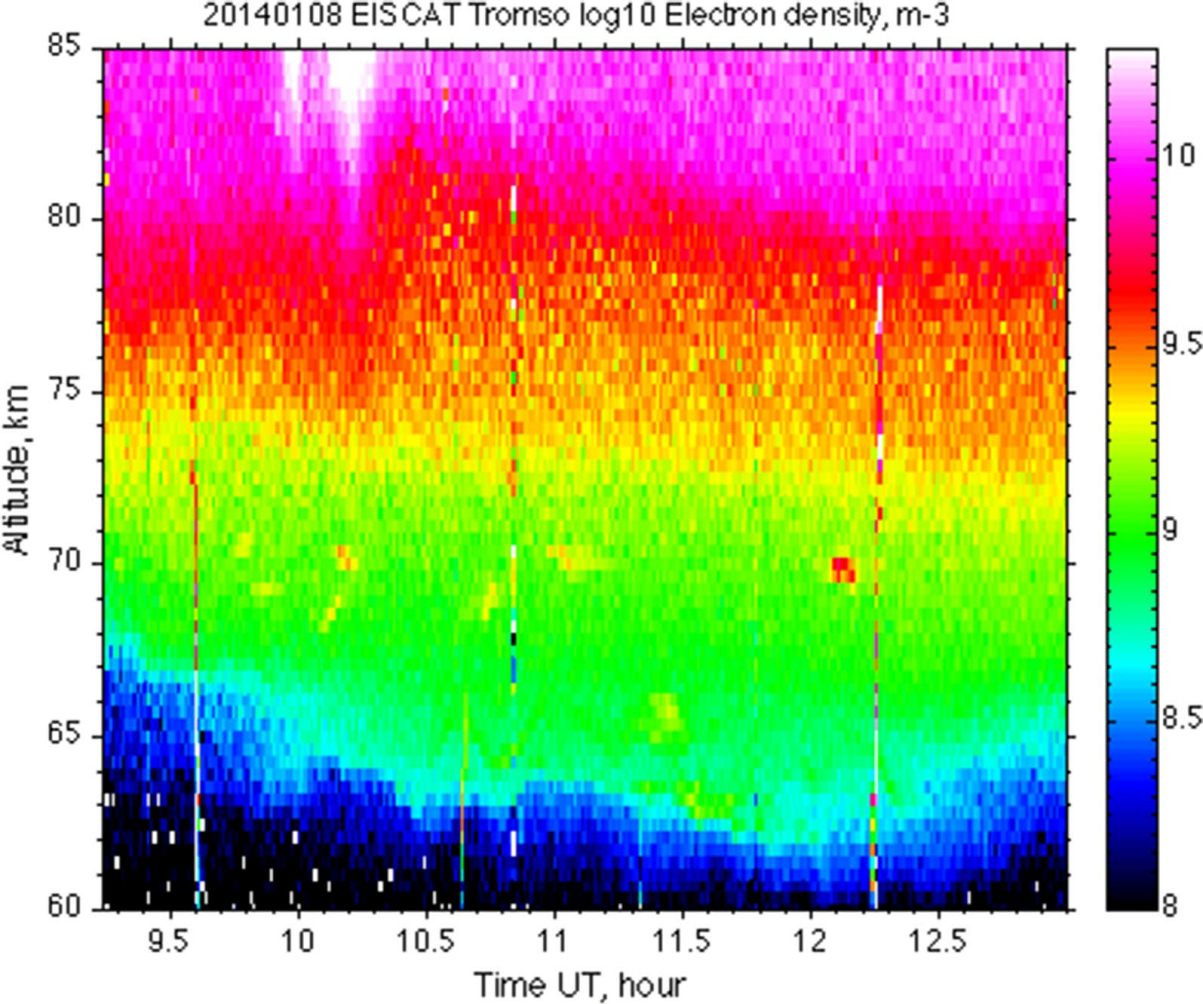
Electron density (with 48 s time integration) derived from the EISCAT VHF radar measurements at Tromsø using GUISDAP

The data for TRO were analysed using the standard analysis package GUISDAP (Lehtinen and Huuskonen 1996). In Fig. 2 the electron density is shown as a function of altitude and time. We see there that the electron density was enhanced from 62 km upward. The reason of this enhancement was solar energetic proton precipitation which ionised the ionosphere. Increased fluxes of solar protons were detected by the GOES13 satellite on 7–9 January (SWPC historical database 2014). Against the background of elevated electron density, there are superimposed several patches of increased scatter at about 70 km altitude. These are polar mesosphere winter echoes (PMWE). Several patches of enhanced backscatter at the lower altitudes (e.g. at about 65 km at 11:25 UT) were found to be airplane clutter as their spectra were very narrow and ‘moved’ quickly away. For the further analysis, we limit ourselves to the interval 11:00–13:00 UT where the strongest echo was observed at about 12:06 UT.

**Fig. 3:**
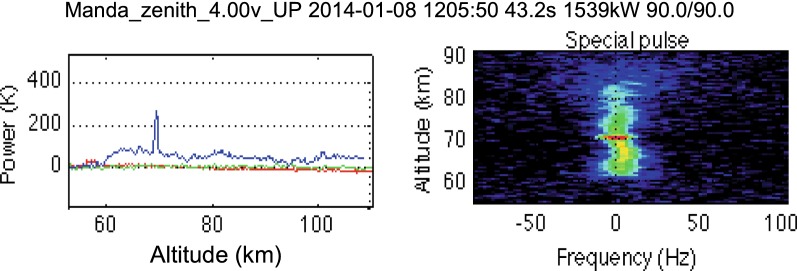
Backscattered power as a function of altitude over Tromsø (left panel, blue curve) and spectra of backscattered signal for different altitudes (right panel) as on the EISCAT real-time graph (RTG). Green and red curves (left panel) are power profiles which are not relevant for this study. Colour scale is an indicator of power spectral density in relative units. The title at the top contains information about experiment name, date, time, post-experiment integration, transmit power, azimuthal and elevation angle of the antenna

Figure 3 shows the altitude profile of backscattered power and signal spectrum derived from the ACFs for the D region for TRO as they are displayed by the EISCAT plotting package real-time graph (RTG) at 12:05:50 UT. We ran RTG after the experiment and applied 43.2 s integration. The backscattered power (blue curve) on the left panel shows a distinct peak at about 70 km altitude indicating PMWE occurrence. The spectrum on the right panel has a peak at the same altitude.

In order to analyse the spectra in more detail and derive spectral parameters, we use the ACFs for the D region recorded every 4.8 s, make integration over several dumps, apply FFT to get spectra and fit with a generalised Gaussian with form:

1 $$S_{\text{fit}} = A \cdot \exp \left\{ { - \,\left( {\frac{{\left| {f - f_{\text{D}} } \right|}}{{\sigma_{f} }}} \right)^{n} } \right\} + A_{\text{N}} = A \cdot \exp \left\{ { - \,\left( {\frac{{\left| {V - V_{\text{D}} } \right|}}{\sigma }} \right)^{n} } \right\} + A_{\text{N}} ,$$

where *A* is a spectral amplitude, *A*_N_ is noise spectral density, *f* is a frequency, *f*_D_ is a Doppler shift, *σ*_f_ and *σ* are quantities proportional to spectral width in frequency and velocity units, respectively, *n* is an exponent, *V* is a velocity, *V*_D_ is a Doppler velocity expressed by the formula:

2 $$V_{\text{D}} = f_{\text{D}} \cdot \lambda_{\text{Bragg}} = \frac{{f_{\text{D}} \cdot \lambda_{\text{r}} }}{{2 \cdot \sin \left( {{\raise0.7ex\hbox{${\theta_{\text{s}} }$} \!\mathord{\left/ {\vphantom {{\theta_{\text{s}} } 2}}\right.\kern-0pt} \!\lower0.7ex\hbox{$2$}}} \right)}}$$

here *λ*_Bragg_ is Bragg’s wavelength, *λ*_r_ is a radar wavelength (1.34 m), *θ*_s_ is a scattering angle. For TRO *θ*_s_ = 180° and *λ*_Bragg_ = 0.5 *λ*_r_ = 0.67 m. However, for the remotes *λ*_Bragg_ = 0.82 and 0.88 m for KIR and SOD, respectively. Electron density fluctuations on the spatial scale equal to the Bragg wavelength for the specific scattering angle make most contribution to the signal received at the site due to constructive interference of the radar incident and scattered waves.

**Fig. 4:**
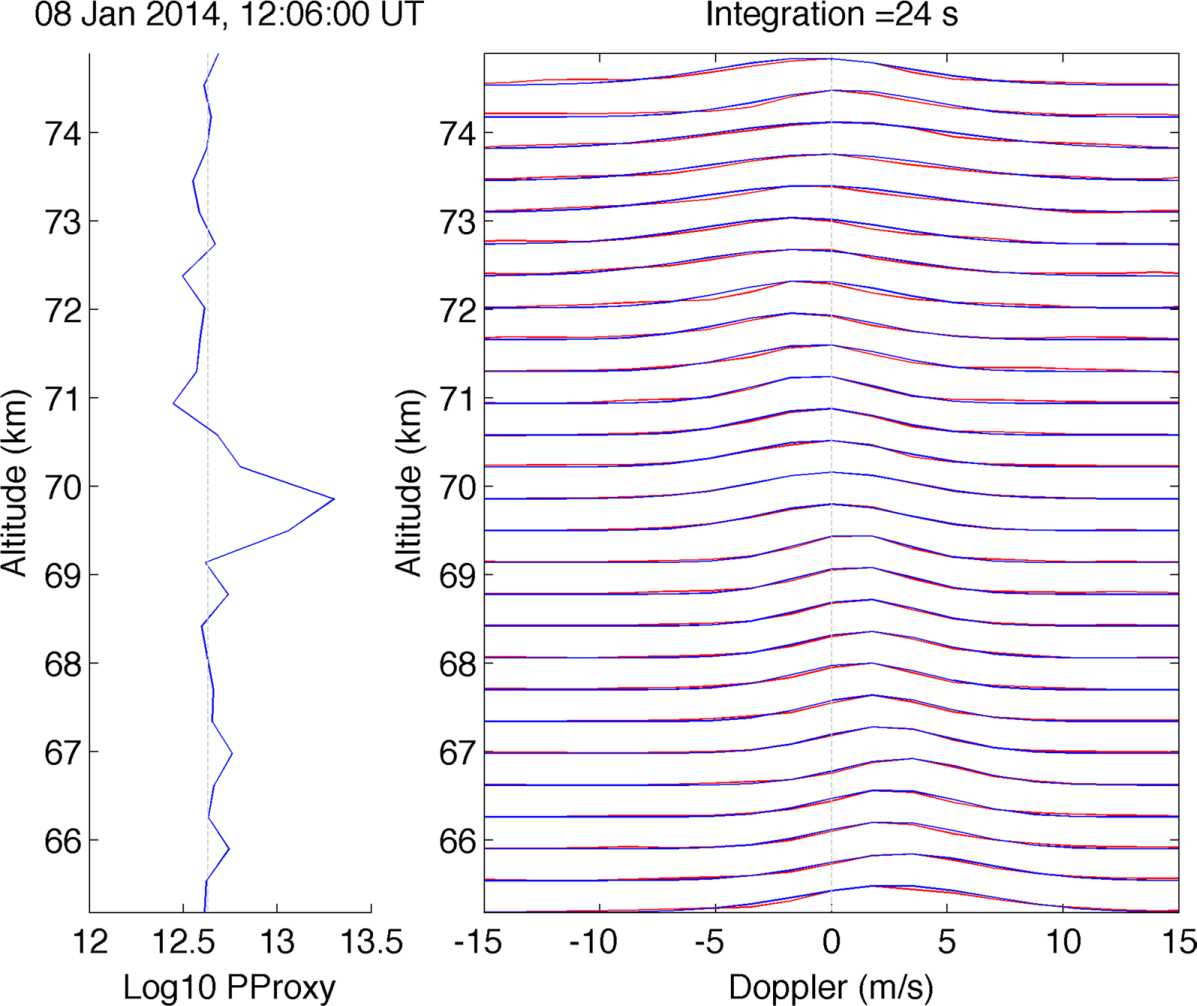
(Right panel) Experimental and fitted spectra for Tromsø at different altitudes at 12:06 UT Spectrum at each altitude are normalised to its maximum. (Left panel) Vertical profile of the logarithm of variable PProxy, proportional to the backscattered power (see the text for more explanations)

An example of the experimental and fitted spectra at different altitudes is presented in Fig. 4 (right panel). The spectrum at each altitude is normalised to its maximum. On the left panel the vertical profile of variable PProxy calculated from the ACF at near-zero lag multiplied by altitude squared is depicted too. This quantity is proportional to the total backscattered power and shows the peak just below 70 km. We see that fitting is in very good agreement with experimental spectra for the whole range of altitudes from 65 to 75 km, i.e. where conventional incoherent scatter (IS) and enhanced echo (PMWE) take place.

**Fig. 5:**
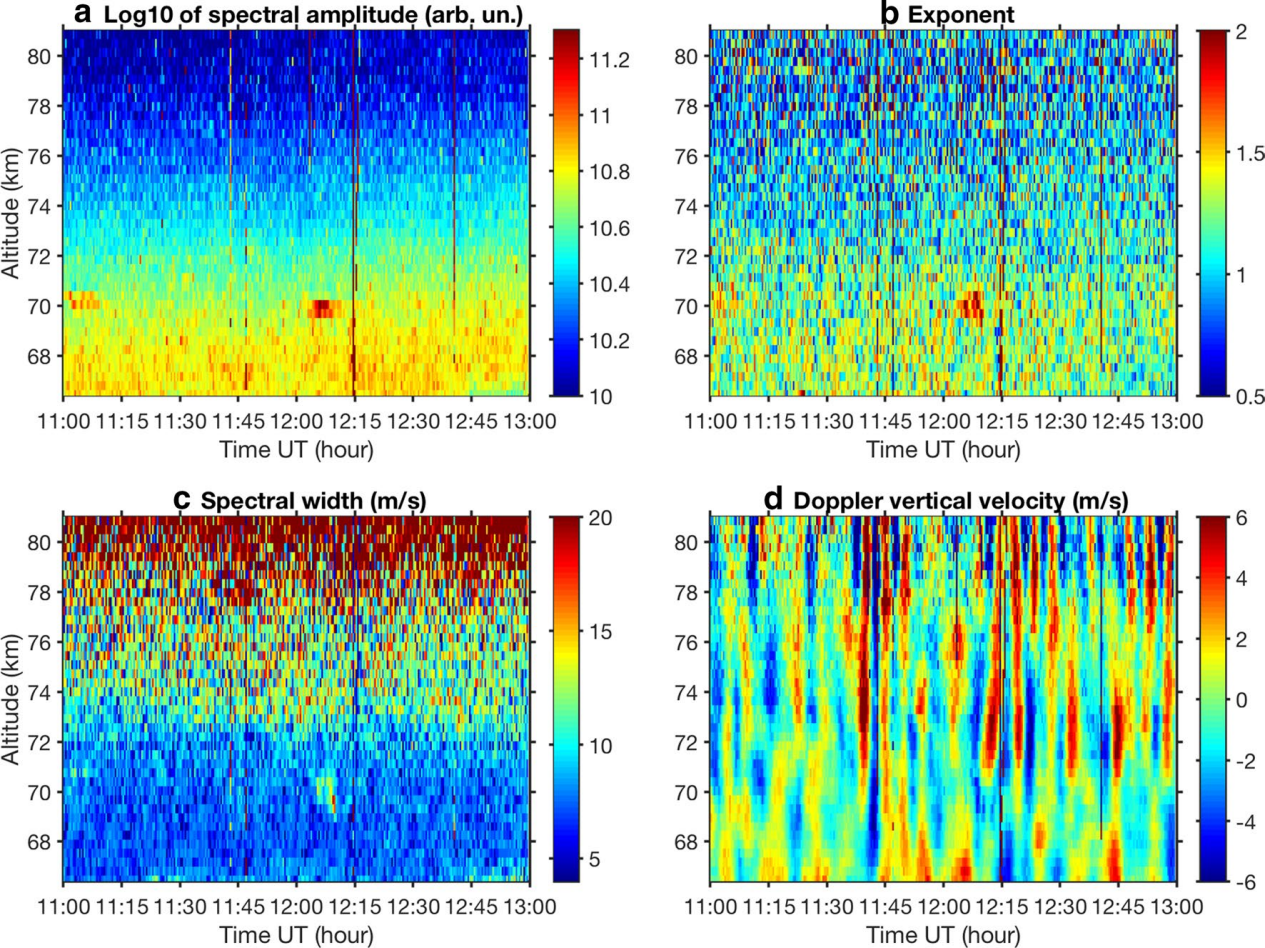
Time–altitude plot of fitted spectral amplitude (**a**), exponent (**b**), full spectral width (**c**) and vertical velocity (**d**) for Tromsø. The (almost) vertical lines at 11:44, 11:46, 12:02, 12:14 and 12:40 UT indicate range aliased echoes of space debris

**Fig. 6:**
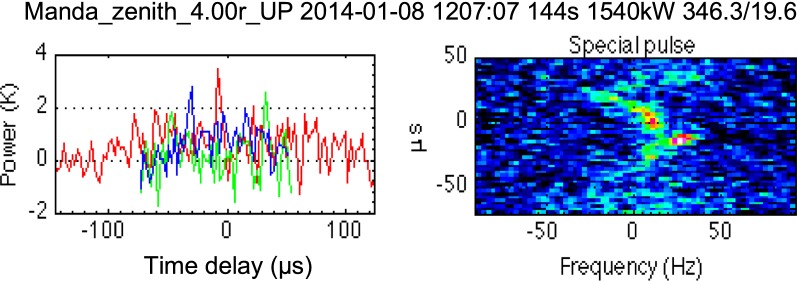
Same as for Fig. 3 but for the receiver in Kiruna. The X-axis on the left panel and Y-axis on the right panel are the signal time delays in μs relative to the signal which comes to Kiruna from the central pointing altitude (75 km)

We applied the fitting for the TRO spectra integrated over 24 s for the time interval 11:00–13:00 UT, and results in terms of spectral amplitude, exponent, Doppler vertical velocity and spectral width are presented in Figs. 5 and 6.

## Experimental results

### Tromsø

In Fig. 5a–d the time–altitude plots for all fitted spectral parameters are presented. The spectral amplitude (Fig. 5a) is largest at the time and altitudes where PMWE occur as we have already seen in Fig. 2. The exponent (Fig. 5b) is larger for PMWE than for other spectra. This is in the agreement with the results by Strelnikova and Rapp (2013) based on 32-h PMWE observations with the EISCAT VHF radar. They found that the ACFs inside PMWE have exponent median value of 1.6, whereas outside the echoes this value is about 1. In general, the ACF is the Lorentzian for IS for the D region (e.g. Mathews 1978) and a superposition of the Gaussian and Lorentzian for PMWE. Then after a Fourier transform we will have spectrum with the exponent less than 2 for PMWE. For the IS spectrum, the exponent will be close to 1.

In Fig. 5c the time–altitude plot for the fitted spectral width is presented. Because a simple relationship between parameter σ and spectral width exists only for the Gaussian and Lorentzian spectra, we derive the actual spectral width at the half maximum level of the fitted spectrum and express it in velocity units using Eq. 2. The spectral width gradually increases with height. This is expected since the IS ion line width is inversely proportional to the ion-neutral collision frequency which decreases with height (e.g. Kofman et al. 1984). The spectra for the winter echoes are slightly wider than those for IS at the same altitudes.

Figure 5d shows the Doppler shift of the radar returns expressed as the speed at which the scatterers are moving vertically towards or away from the radar. In the highly collisional D region the observed scatterer travel speed can be interpreted as corresponding to the vertical component of neutral wind. The figure reveals strong vertical motion with up to a few m/s speed, which changes more or less regularly from positive to negative values. The mean vertical velocities are believed to be a few cm/s in the mesosphere (Holton 1983; Garcia and Solomon 1985). The observed velocities can be interpreted as the neutral winds modified by gravity waves. One can distinguish three altitude regions where waves are different with respect to their behaviour. Below 70 km the waves are not so strong and regular. More distinct waves with periods of 5–10 min are seen between 70 and 77 km starting from ~ 11:40 UT. Then from 77 km upward, waves with 5-min period prevail. Interestingly, at ~ 70 km where the PMWE patches were observed, the waves have amplitude of about zero. The presence of such waves has to be taken into account in analysis of echo spectra for the remote sites.

### Kiruna and Sodankylä

Our next step was to analyse remote sites KIR and SOD in order to see if PMWE were detected there too. Firstly, we made a visual inspection of the spectra of the signal received at KIR and SOD using the RTG package and found that enhanced echoes were observed at the same time as the strongest echo at TRO (about 12:05–12:11 UT). Figure 6 shows one example of the RTG plots for Kiruna at 12:07:07 UT. Here the power (left panel) and spectra (right panel) are shown as a function of travel time instead of altitude as in Fig. 3 for TRO. 0 μs corresponds to travel path: TRO-75 km altitude above TRO-KIR; then there are 26 gates with shorter (negative) travel times and 26 gates with longer (positive) times with 2.4 μs gate separation as described in "Experiment description" section. KIR signal is much weaker and noisy compared to TRO; therefore, a longer time integration of 144 s was applied for KIR. (SOD signals are even weaker than KIR ones.) The main reason for this is that received power is inversely proportional to signal path length squared, which is longer for KIR. Doppler shifts of spectra for KIR are larger than those for TRO, because they are related to the horizontal component of wind rather than vertical one as for TRO and this will be discussed later.

Spectra for remote sites can experience significant shear broadening (Hocking 1983), which depends on their line-of-sight and, hence, is different for KIR and SOD. Therefore, we decided to limit ourselves to derivation of the Doppler velocities for the remotes. Remote receivers are sensitive to the plasma drift velocity (= neutral wind in the D region) component along the bisector of the angle between the transmitter and receiver beams. The geometry of our experiment was the same as described by Janches et al. (2002) in Fig. 9. Because our scatterers move with the neutral wind whose horizontal component is much larger than the vertical one (e.g. Holton 1983), KIR and SOD measure mainly horizontal wind components along TRO-KIR and TRO-SOD lines, respectively. Meridional and zonal winds can finally be derived by combining KIR and SOD Doppler shifts.

**Fig. 7:**
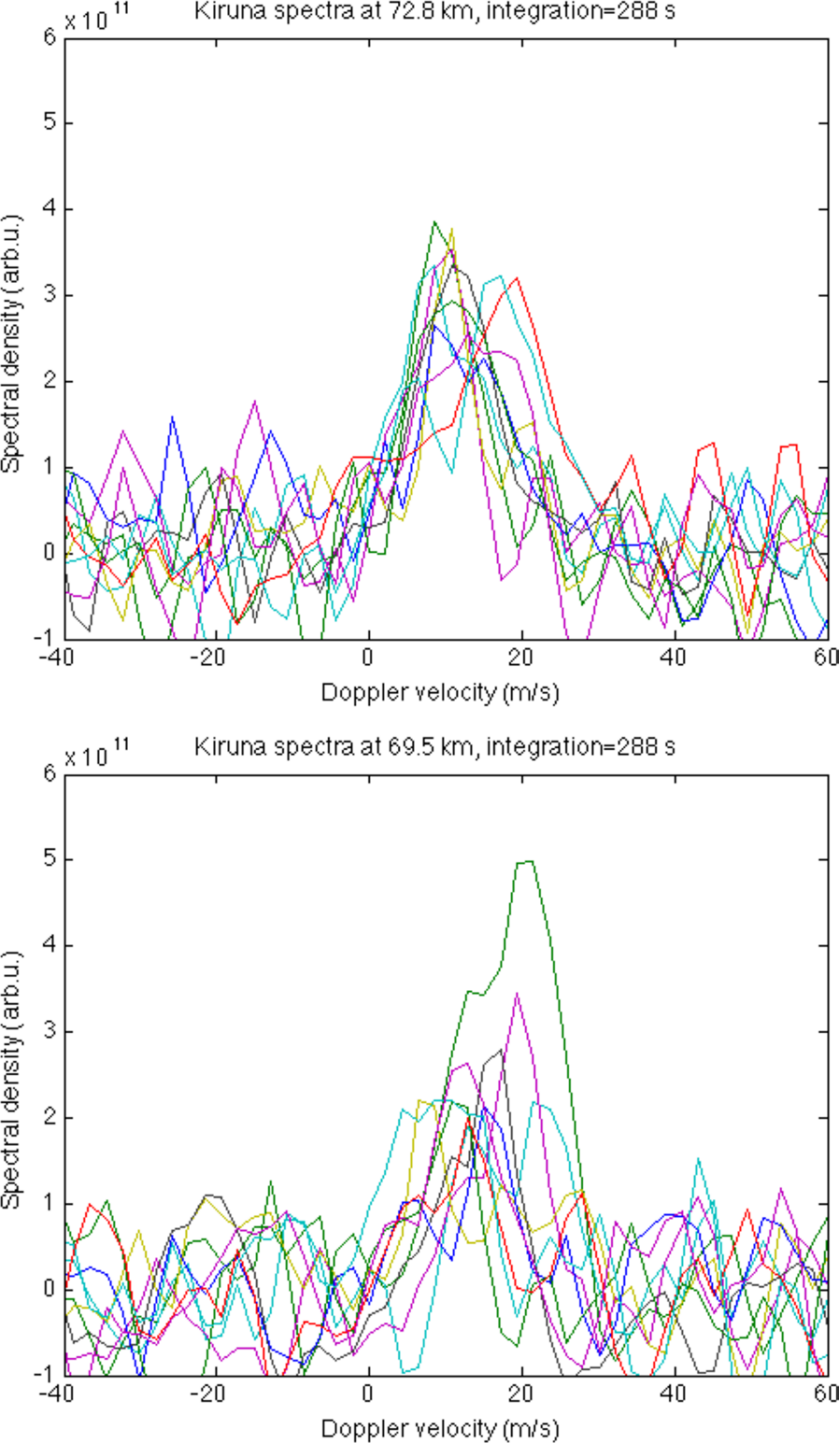
Experimental spectra of the signal received in Kiruna from 72.8 km (upper panel) and 69.5 km (lower panel) altitude for 12:00–13:00 UT at different times separated by 288 s (indicated by different colours)

We computed the experimental spectra for KIR from the ACFs in the same way as for TRO. The travel times were converted to altitudes (see Table 1). In order to get spectra of satisfactory quality, we have to make the time integration of ACFs long enough. The longest integration we can apply is about 5–6 min, determined by the PMWE duration. Additionally, when applying integration, one should remember that strong waves with 5–10 min periods were present there as seen in Fig. 5d. When we had applied relatively short integration of tens of seconds, the resulted spectra were still too noisy to be successfully fitted by the model function according to Eq. 1. However, when integrating over longer time (a few minutes) we obtained better spectra but very often they comprise of two nearly equal peaks. Then fitting by one Gaussian does not give good results. One example for KIR for two altitudes with 288 s integration is shown in Fig. 7. There the spectra at 72.8 km have about the same peak magnitude over the course of time, while those at 69.5 km have a clear maximum at the time when PMWE were observed in TRO. The applied integration eliminates the effect of waves to some extent, however not completely.

**Fig. 8:**
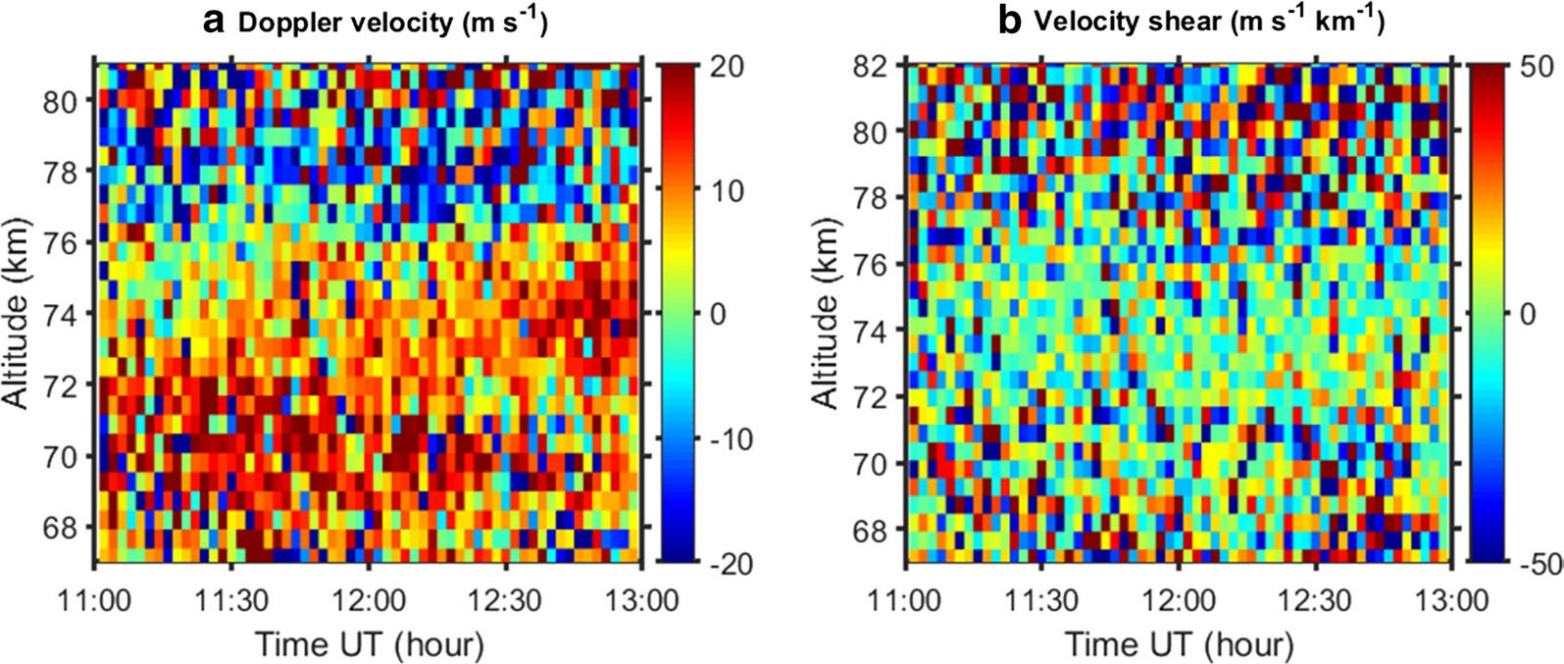
Time–altitude plots of Doppler velocity (**a**) and velocity shear (**b**) for the Kiruna receiver

Because of the noisy spectra, we decided to apply another method for derivation of Doppler velocities from KIR and SOD data, which uses ACF instead of spectrum. Then we avoid the Fourier transform and, hence, additional computational errors. We used the ‘matched filter’ method described by Evans et al. (1970). It is based on trials and was used for determination of vertical drifts in the F region where both ACF and IS spectrum have a sophisticated shape. (The spectrum is double peaked.) In Fig. 8 time–altitude plots for the Doppler velocity as well as the Doppler velocity shear averaged over 120 s (25 dumps) are presented. We see here that during the interval from 11:00 until about 12:50 the velocity is positive at the lower heights with maximum at about 70–72 km altitude. From about 77 km, the velocity changes direction and becomes mostly negative at higher altitudes. The velocity shear looks noisy, and it is small at most times and heights with some enhancement in magnitude at about 71 km altitude. Neither velocity and shear show anything special in their behaviours at the time and height where PMWE were observed in the TRO (Fig. 4) and KIR spectra (Fig. 7). This implies that the wind shear exists during the entire 2-hour interval 11:00–13:00 UT and has no relation to the PMWE occurrence.

**Fig. 9:**
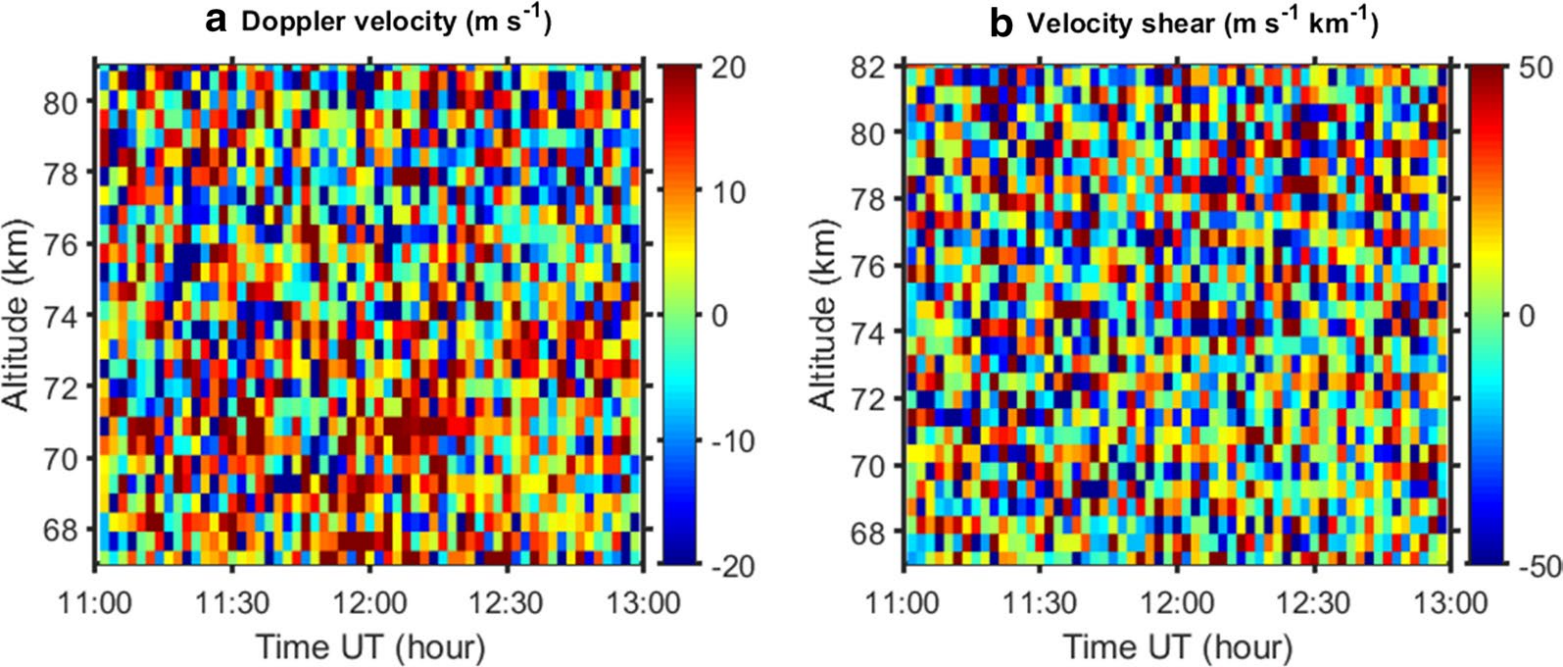
Same as Fig. 8 but for Sodankylä

The ‘matched filter’ analysis with the same integration over 120 s was done for SOD, and the results are shown in Fig. 9. The spectral maximum (not shown) is at least 2 times less than for KIR, the Doppler velocity and especially its vertical shear show significant variations with altitudes and time. What we can notice is a patch of the high positive velocity at 70–71 km altitude at 12:05–12:20 UT which somehow matches the PMWE time and location. Such strong variation for the Doppler velocity and shear can be explained by the weaker received signal resulting in very noisy ACFs. Additionally, for SOD the scatter signal comes from a larger height range than for KIR (see Table 1).

## Discussion

In this paper, we determined the Doppler shifts for TRO using fitting the spectra calculated from ACFs measured in the radar experiment. For the weaker signals for KIR and SOD, we derived the Doppler velocities directly from the ACFs using the ‘matched filter’ method. We have applied this method to the TRO data as well and the results (not shown) were the same as those from spectral fit (Fig. 4d). The spectral fit method used for TRO additionally gives us the spectral width and exponent which are useful for further interpretation of the echo mechanism.

During interval 11:00–13:00 UT we observed strong gravity waves with periods of 5–10 min in vertical velocity derived from the TRO data. The most strong and regular waves are confined between ca 70 to 80 km where their phase does not show significant changes with height. This behaviour is a feature of ducted waves. Similar ducted waves were previously observed by the EISCAT VHF and SOUSY (53.5 MHz) radars (Fritts et al. 1990; Hoppe and Fritts 1995). The duct can be formed around a horizontal wind maximum (e.g. Fritts and Yuan 1989). We observed such maxima in the Doppler velocity magnitude at about 70 and 78 km altitude with the KIR receiver (Fig. 8a). For the receiver in Sodankylä, the maximum in the Doppler can be seen less obviously at 71 km during 15 min from about 12:07 UT (Fig. 9a).

Enhanced radar echoes (PMWE) were observed with all three receivers in TRO, KIR and SOD at about 70 km altitude as a patch of ca 1 km thickness lasting 5–6 min. At ionospheric altitudes the radar electromagnetic wave is scattered from perturbations in the electron density of a specific scale (the radar Bragg scale). As was discussed in previous studies (e.g. Lübken et al. 2006; Belova et al. 2013) for the PMWE case, neutral turbulence with or without charged dust might be a cause of such perturbations. In our experiment the PMWE altitude coincides with the lower border of the duct and with the altitude where the enhanced horizontal wind shear occurs. Recently, wind shear was found in relation with polar mesosphere summer echoes (PMSE) using the tristatic EISCAT VHF radar (Mann et al. 2016). Then the authors suggested that Kelvin–Helmholtz (KH) instability plays a role in PMSE formation. However, our observations did not reveal any relation between enhanced echo occurrence and the strength of the wind shear (Fig. 8b). Probably, the value of the wind shear is not enough for excitation of KH instability resulting in enhanced turbulence, which can lead to PMWE generation. KH instability occurs when the Richardson number R_i_ is in range between 0 and 0.25. R_i_ is defined by formula:

3 $$R_{i} = \frac{{N^{2} }}{{\left( {wind\_shear} \right)^{2} }},$$

where buoyancy frequency *N* squared is characteristic of static stability of the air and the term in the denominator is horizontal wind shear.

The possible trigger of KH instability might be some additional wind or *N*^2^ fluctuations which cannot be detected with the EISCAT radar; both can occur, e.g. due to small-scale waves. There are other relevant instruments at the TRO site to measure wind and temperature profiles: a MF-radar (University of Tromsø) and a sodium lidar (Nagoya University, Japan), respectively. Unfortunately, both instruments did not have measurements useful for our case: the former had no reflections from the PMWE altitude range during the time interval of interest and the latter did not operate due to overcast conditions.

Another mechanism producing turbulence is breaking gravity waves. Rapp et al. (2011), using multi-beam observation with the MAARSY radar, concluded that the 3D behaviour of PMWE was reminiscent of breaking gravity waves with period of about 4 h. The authors found that the enhanced echoes followed the wavefront of the westward propagating gravity wave. In our case, the strong gravity waves with much shorter periods of 5–10 min were detected in vertical wind. We did not see such waves in the horizontal components of wind derived from KIR and SOD because the signal was too weak and too noisy at the remote sites. Another possible reason is that the amplitude of these waves was significantly smaller than the horizontal winds themselves. Importantly, the behaviour of the vertical wind at 66–81 km altitudes at least after 11:40 UT is characteristic for ducted rather than for propagating waves. If we speculate that these waves are propagating upward, breaking and producing turbulence at 70 km altitude which lead to PMWE generation why does this mechanism work only once at 12:05? Also, the weaker PMWE at about 11:00 UT does not coincide with any break in the wave signature at that height/time.

Another possibility for PMWE generation is the presence of patchy charged dust that leads to increased gradients in electron density and, hence, to enhanced radar backscatter. The dust is smoke particles of nm size originating from meteoroid ablation in the altitude range between 70 and 110 km (e.g. Hunten et al. 1980). These particles become charged due to attaching electrons or due to photoionisation. The presence of charged particles of a few nm radii during PMWE events was confirmed by ionospheric modification experiments using the EISCAT Heating facility (Belova et al. 2008; La Hoz and Havnes 2008; Havnes et al. 2011).

We also estimated spectral width for TRO measurements in the presence and absence of PMWE. Radar echoes in the D region are subject to different spectral broadening, e.g. beam, shear and wave broadening. Waves with periods 5–10 min detected there (Fig. 5d) do not contribute in spectral width calculated with 24 s time integration. Shear broadening is important for tilted beams. TRO spectral width *w* should, first of all, be corrected for beam broadening *w*_b_ as: (w^2^ − *w*_b_^2^)^0.5^. Here the spectral width broadening due to finite beam *w*_b_ can be estimated as (Hocking 1985):

$$w_{{{\text{b}} }} \left( {{\text{m}}/{\text{s}}} \right) \approx 0.35 \cdot \theta \cdot V_{\text{hor}} ,$$

*θ* is the 3 dB full beam width in radians and *V*_hor_ is the horizontal wind speed. Taking *V*_hor_ to be equal to 70 m/s which is the maximum of the horizontal wind observed at 70 km by the MF-radar in Tromsø during the entire day of 8 January, one can get *w*_b_ ~ 2 m/s as a conservative estimate. This relatively small spectral broadening will not significantly affect the measured spectral width of 5–10 m/s.

We found that the spectral width of PMWE in the vertical beam was larger than that in their absence, i.e. than for IS spectra (8–10 vs. 5–6 m/s), with any beam broadening expected to contribute equally to both. This is in line with the result reported by Belova et al. (2013) for winter echoes during a solar proton event in November 2004 although spectral width values were different there. This may be interpreted as enhanced neutral turbulence or/and presence of patches of small negatively charged meteoric dust. The former results in increased turbulent velocities of neutrals and, hence, in highly collisional plasma, ions and electrons, that we see as enhanced spectral width. According to the model calculations depicted in Figs. 10 and 11 of Belova et al. (2013), in order to have spectral widths as measured on 8 January 2014 the turbulence dissipation rate should be in the range between 150 and 500 mW/kg. This is very high compared to the typical values of 1–10 mW/kg derived from the numerous rocket experiments for 60–75 km in wintertime (Lübken 1997). The presence of negatively charged particles with radius less than 1 nm leads to increasing electron diffusivity (as negative ions do in the lower D region) that widen the IS spectra (Cho et al. 1998) and could explain our observations. However, the mechanism that confines meteoric dust in narrow layers or patches is still unknown.

Kirkwood et al. (2006b) proposed non-turbulent mechanism for generation of very strong PMWE, which involves infrasound waves propagating from below. Then the echoes would move horizontally with the speed of sound. It does not seem to be the case for the PMWE on 8 January 2014, where the velocity components measured at the remote sites were up to 25 m/s and the echoes were not very strong.

## Conclusion

Small patches of PMWE were observed during solar proton event on 8 January 2014 using the tristatic configuration of the EISCAT VHF radar. The strongest echo was detected from about 70 km altitude at 12:05–12:11 UT, and the interval 11:00–13:00 UT was analysed in more detail. For the TRO data we fitted experimental spectra by a generalised Gaussian function and analysed its parameters. For the KIR and SOD data, we used experimental ACFs to derive horizontal velocities.

Gravity waves with periods of about 5–10 min were found in the vertical velocity using Tromsø data. They behave as ducted waves in the waveguide between 70 and 78 km. The duct might be formed around the horizontal velocity maxima observed at these heights with the receiver in Kiruna.

We did not find any relation of the PMWE occurrence with the strength of the horizontal wind shear observed at the same heights. We argue that this shear, probably, is not large enough for excitation of KH instability/enhanced turbulence which can lead to PMWE. The possible trigger might be additional small-scale waves which cannot be detected by the EISCAT radar. The results obtained with this tristatic radar experiment do not allow us to completely rule out the breaking gravity wave mechanism. Another possible mechanism for the echo generation which can qualitatively account for our radar observations is the presence of patchy charged dust such as meteoric smoke particles.

For PMWE observed at TRO, the spectral width was enhanced compared to that for the incoherent scatter at the same heights. We interpret this as rather an indication of patches of negatively charged small-sized dust than enhanced neutral turbulence as the turbulence levels required would be unreasonably high.

We can conclude that tristatic radar measurements are shown to be a useful method to study PMWE and their generation mechanism. We have only a few minutes of winter echo observations so far. In order to shed a light on the origin of PMWE, more experimental data during different ionospheric and echo conditions are needed. We plan to make more tristatic experiments in future using the EISCAT radars.

## Abbreviations

ACF: autocorrelation function
EISCAT: European incoherent scatter
IS: incoherent scatter
KH: Kelvin–Helmholtz
KIR: Kiruna
PMSE: polar mesosphere summer echoes
PMWE: polar mesosphere winter echoes
RTG: real-time graph
SOD: Sodankylä
TRO: Tromsø
